# Impact of depression, anxiety, and COVID-19 diagnosis on social isolation trajectories during the pandemic: A 3-year prospective cohort study

**DOI:** 10.1371/journal.pone.0330118

**Published:** 2025-09-10

**Authors:** Juhee Choi, Gaeun Son, Ye-Seul Kim, Kee-Hong Choi, Jungeun Kim, Subin Park

**Affiliations:** 1 School of Psychology, Korea University, Seoul, Republic of Korea; 2 KU Mind Health Institute, Korea University, Seoul, Republic of Korea; 3 Division of Research Planning, Mental Health Research Institute, National Center for Mental Health, Seoul, Republic of Korea; 4 Mental Health Research Institute, National Center for Mental Health, Seoul, Republic of Korea; Wuhan Mental Health Centre, CHINA

## Abstract

**Background:**

The coronavirus disease 2019 (COVID-19) pandemic has profoundly affected physical and mental health. Since the onset of the pandemic, the prevalence of depression and anxiety has significantly increased. Quarantine and social distancing, implemented to control the spread of COVID-19, have exacerbated social isolation. This study aimed to longitudinally examine how the severity of depression and anxiety, along with COVID-19 diagnosis, influenced the trajectory of social isolation throughout the pandemic.

**Method:**

This longitudinal study collected data from South Korea in three waves: 2021, 2022, and 2023. The final sample included 2,395 participants (mean age = 46.32, SD = 16.47) who completed a face-to-face interview survey. Depressive symptoms were measured using the Patient Health Questionnaire-9, while anxiety was assessed using the Generalized Anxiety Disorder-7. Social isolation was evaluated using the Loneliness and Social Isolation Scale. The data were analyzed using multilevel modeling with a three-way interaction design.

**Results:**

A significant three-way interaction was found between time, depression severity, and COVID-19 diagnosis, while anxiety severity showed no significant interaction. Among the participants diagnosed with COVID-19, those with severe depressive symptoms exhibited an increase in social isolation over time. The positive effect of time was strengthened when the depression severity was severe, whereas it was weakened in participants with moderate or mild depressive symptoms. For participants without a COVID-19 diagnosis, social isolation tended to decrease over time across all levels of depressive symptoms, excluding cases with no depressive symptoms.

**Conclusions:**

The three-way interaction results indicated that the severity of depressive symptoms and COVID-19 diagnosis status significantly influenced social isolation trajectory over time. Individuals diagnosed with COVID-19, particularly those experiencing severe depression, have an increased risk of worsening social isolation over time. This underscores the need for intensive and sustained psychological support and intervention for vulnerable individuals.

## Introduction

The first confirmed case of coronavirus disease 2019 (COVID-19) occurred in China in December 2019, and a pandemic was declared in March 2020 as it spread worldwide. As the pandemic extended beyond 2 years, its prolonged impact on daily life has increasingly threatened both physical and psychological well-being worldwide [1,2].

Numerous studies have reported a deterioration in mental health during the COVID-19 pandemic, with elevated levels of depression, anxiety, posttraumatic stress, and suicidal ideation observed in the general population [3,4]. Among these, depression and anxiety have been particularly prevalent. A meta-analysis revealed a significant global increase in these disorders during the pandemic [5]. According to the World Health Organization, the global prevalence of depressive and anxiety disorders rose by approximately 25% in the first year of the pandemic [6]. Empirical studies further reported a depressive symptom prevalence ranging from 32.0% to 35.8% [7]. In South Korea, a population-based study found that 30.7% and 22.6% adults were at risk for depression and anxiety, respectively; these rates were substantially higher than pre-pandemic rates [8].

Notably, these mental health problems were worse in COVID-19 cases than in non-confirmed cases [8]. Only 8.8% of patients with COVID-19 had no psychiatric symptoms, 91.2% had psychiatric symptoms [9], and COVID-19 survivors had significantly higher rates of anxiety and depression [2,10].

In the early stages of COVID-19, social distancing policies were implemented worldwide to control the spread of the infectious disease [11]. In Korea, policies included mandatory quarantine, lockdowns, remote work, and restrictions on private gatherings [12]. These measures significantly weakened social networks and disrupted interpersonal relationships, contributing to heightened psychological vulnerability [13]. A meta-analysis reported that the pooled prevalence of social isolation was 31.2% during the pandemic—markedly higher than the pre-pandemic estimates—underscoring the widespread impact of social disconnection on mental health [14]. Increased levels of loneliness were also observed in countries such as China and the United Kingdom, particularly after all restrictions were implemented [15,16].

Social isolation and mental health appear to have a bidirectional and self-reinforcing relationship. Individuals experiencing psychological distress often withdraw from social interaction, further exacerbating feelings of isolation and interfering with recovery [17,18].

Previous studies highlighted the impact of the pandemic on social isolation and mental health [19,20]. However, the relationship between other mental health problems and social isolation was only examined using cross-sectional designs. Moreover, much of the existing literature focuses on the link between social isolation and mental health in the senior population and considers this population to be vulnerable [21,22]. However, a review involving children and adolescents has also suggested a significant impact of social isolation on mental health in younger groups [23]. A few studies have reported results for younger age groups, although they focused on hospitalized patients with cancer and their caregivers [24] or university students, rather than the general population [25,26].

Collectively, COVID-19 has affected mental health including depression, anxiety, and social isolation. However, most studies examined the relationships between mental health problems and social isolation using a cross-sectional design and specific age groups.

Given these limitations, further research is needed to clarify how depression and anxiety contribute to social isolation over time and to determine whether this process is moderated by the COVID-19 infection status. To address this gap, the present study aimed to

(a) investigate how baseline levels of depression and anxiety predicted changes in social isolation over a 3-year post-pandemic period and
(b) examine whether the relationship between mental health and social isolation was moderated by a history of COVID-19 diagnosis.

## Methods

### Sample and design

The COVID-19 Mental Health Panel Survey was conducted to address the research questions. The COVID-19 Mental Health Panel Survey aimed to investigate the current status of and changes in mental health problems caused by COVID-19 among the public in South Korea. The sampling frame was based on those who were aged 15–79 years, as provided in the Population and Housing Census at the time of the design. The study was approved by the Institutional Review Board of the National Center for Mental Health Institutional Review Board (IRB No.116271-2021-30). Before the interviews, each respondent was informed of the objectives and methods of the survey. Each respondent signed an informed consent form collected through a tablet PC. When minors (15–17 years old) were included in this survey, informed consent was also obtained from their parents or legal representatives.

When designing the survey, the sample size was determined to be 1,800 non-COVID-19 confirmed cases and 600 confirmed cases, considering the representativeness of the population and comparison between COVID-19 confirmed cases. The samples were stratified according to region, sex, and age. Because the prevalence of COVID-19 cases in South Korea was very low at the time of the 2021 survey (approximately 0.8%) [12], survey investigators visited the sample households and directly inquired about any confirmed COVID-19 cases within the households visited. If the household included a member with confirmed COVID-19, that member was included in the survey. If there were no COVID-19 cases in the household, the next participant was selected by probability proportional to size sampling. The participants were asked the following question: “Have you ever been confirmed to have COVID-19?” If they responded “yes,” we investigated the date of confirmation by the hospital or public health center. Accordingly, a diagnosis of COVID-19 was defined as a diagnosis of COVID-19 confirmed at least once by 2021. Finally, 1,963 non-confirmed cases and 640 confirmed cases were recruited in 2021.

The COVID-19 Mental Health Panel Survey began annual data collection in 2021 and utilized data up to the third year. It recruited 2,603 people in the first year of 2021, bringing together 2,510 people in 2022 and 2,462 in 2023, reporting a 94.6% retention rate.

Because this panel survey was a face-to-face interview survey, 126 investigators were trained before conducting the survey. The training included an overview of the survey process, instructions on the survey form, tablet-assisted personal interviewing (TAPI), and practical exercises. Only the investigators who completed the entire training program were assigned to the field. Given the longitudinal nature of a panel survey, which requires the same participants to be surveyed annually, the same investigator conducted the survey as much as possible. The survey was conducted annually from September to December. Using the designed survey tool, an experienced investigator visited the home in person and conducted an interview using a tablet PC.

### Variables

Social isolation was assessed using the Loneliness and Social Isolation Scale (LSIS). The LSIS was developed in accordance with the Korean context to measure the degree of loneliness and social isolation. The scale consists of six questions, and respondents respond to each question on a 4-point scale. The scale comprises three subfactors: loneliness (the subjective aspect of social isolation; two questions), social support (the functional aspect of social isolation; two questions), and social network (the structural aspect of social isolation; two questions). The score was calculated by summing each score: the larger the sum, the more loneliness you feel, the less social support you receive, and the smaller the size of the social network. Although these subcomponents are conceptually distinct, prior validation studies support the use of a total score representing a higher-order construct of social isolation. Following this framework and previous research, we used the total LSIS score as an overall indicator of social isolation in this study and have referred to it as “social isolation” throughout [27]. Depression and anxiety scores were used as continuous variables and were measured using the Patient Health Questionnaire-9 (PHQ-9, total score of 27) [28] and Generalized Anxiety Disorder Questionnaire-7 (GAD-7, total score of 21) [29], respectively. The PHQ-9 cutoff scores, based on Park et al. [30], were categorized as no symptoms (0–4), mild (5–9), moderate (10–19), and severe (20–27). The GAD-7 cutoff scores, following Spitzer et al. [29], were categorized as no symptoms (0–4), mild (5–9), moderate (10–14), and severe (≥ 15).

COVID-19 diagnoses were confirmed by querying participants with the question, “Have you ever been infected with COVID-19?” and identified people infected with COVID-19 in 2021, 2022, and 2023 by confirmation date. The demographic characteristics of the participants were recorded. Demographic characteristics used in this study included sex, age, educational status, and economic status. Sex was categorized as male or female. Age was divided into six different categories: 15–19, 20–29, 30–39, 40–49, 50–59, and 60–79. Educational status was divided into five categories: Elementary school or less, Middle school, High school, College or Master’s enrolled, and College or Master’s graduate. The average monthly household income before tax was used to classify the economic status into four categories (million KRW): < 1.5, 1.5–3, 3–5, and >5.

### Statistical analysis

Descriptive analyses were conducted to assess the baseline characteristics of all predictors and evaluate group differences based on COVID-19 diagnosis, employing the chi-square test and *t*-test using SPSS version 26.0. Subsequently, correlation analysis was performed to identify any multicollinearity among the predictors. Multilevel modeling with a three-way interaction design was performed using R version 4.4.1. Model selection was determined by calculating the Akaike Information Criterion (AIC), Bayesian Information Criterion (BIC), and adjusted BIC (aBIC). After determining whether to apply a fixed or random intercept model for time, the model was structured with eight individual-level predictors at the first level, two symptom severity factors (depression and anxiety severity) at the second level, and COVID-19 diagnosis at the third level. Missing data were handled using a listwise method. Participants (n = 2,462) who responded to all three periods were analyzed in the present study. Data from 67 individuals with missing income variable values were excluded, resulting in a final sample size of 2,395 individuals included in the analysis.

## Results

### Preliminary analyses

Little’s Missing Completely at Random (MCAR) test (χ^2^(7) = 12.606, *p* = 0.082) revealed that the missing data were random. Chi-square and ANOVA tests were conducted to determine differences between participants who withdrew during the study and those who completed all 3 years of the study. Chi-square analysis revealed no significant differences in the incidence of COVID-19 diagnoses among participants who completed the study, those who dropped out at T2, and those who dropped out at T3 (*p* = 0.135). Similarly, ANOVA found no significant differences in baseline levels of depression, anxiety, and social isolation among these groups (depression: *p* = 0.116, anxiety: *p* = 0.213, social isolation: **p* *= 0.125). These findings suggested that the baseline variables did not differ significantly between participants who completed the study and those who dropped out.

### Baseline characteristics

A total of 2,395 participants were included in the analysis. At T1, 608 participants (25.4%), representing nearly a quarter of the sample, were diagnosed with COVID-19. The number of participants diagnosed with COVID-19 increased to 1,272 (53.1%) at T2 and 1,413 (59.0%) at T3, accounting for more than half the sample at both time points. To examine differences in demographic characteristics between two groups based on COVID diagnosis in 2021, chi-squared (χ²) tests were used for categorical variables, and independent samples *t*-tests were conducted for continuous variables. Table 1 presents the results of the study.

**Table 1:** Descriptive analysis of predictors and outcome variables for the overall sample and those with COVID-19 diagnosis by 2021.

Three-level predictors and outcome variable		Overall sample (n = 2395)	COVID-19 diagnosis by 2021			
			Yes (n = 608)	No (n = 1787)	Group differences	
					χ^2^	*t*
**Level 1: Individual-level predictors**						
**Sex**	Male	1158 (48.4)	**271 (44.6)**	**887 (49.6)**	4.658^*^	
	Female	1237 (51.6)	337 (55.4)	900 (50.4)		
**Age**	15-19	143 (6.0)	41 (6.7)	102 (5.7)	4.313	
	20-29	348 (14.5)	88 (14.5)	260 (14.6)		
	30-39	354 (14.8)	89 (14.6)	265 (14.8)		
	40-49	455 (19.0)	117 (19.2)	338 (18.9)		
	50-59	489 (20.4)	135 (22.2)	354 (19.8)		
	60-79	606 (25.3)	138 (22.7)	468 (26.2)		
**Education status**	Elementary school or less	173 (7.2)	32 (5.3)	141 (7.9)	8.439	
	Middle school	243 (10.1)	52 (8.6)	191 (10.7)		
	High school	1044 (43.6)	282 (46.4)	762 (42.7)		
	College/Master’s enrolled	897 (37.5)	234 (38.5)	663 (37.1)		
	College/Master’s graduate	38 (1.6)	8 (1.3)	30 (1.7)		
**Economic status**	Less than 1.5 million KRW	303 (12.7)	59 (9.7)	**244 (13.7)**	17.578^**^	
	1.5-3 million KRW	607 (25.3)	152 (25.0)	455 (25.5)		
	3-5 million KRW	812 (33.9)	190 (31.3)	622 (34.8)		
	Over 5 million KRW	673 (28.1)	207 (34.0)	466 (26.1)		
**Level 2: Mental health predictors**						
**Depression severity**	T1	2.61 ± 3.73	3.04 ± 4.23	2.47 ± 3.54		3.008^**^
**Anxiety severity**	T1	2.21 ± 3.41	2.62 ± 3.80	2.07 ± 3.25		3.188^**^
**Level 3: COVID Diagnosis-level predictors**						
**2021 COVID Diagnosis**	Yes	608 (25.4)	–	–		
	No	1787 (74.6)	–	–		
**Outcome variable**						
**Social Isolation**	T1	5.59 ± 2.69	5.34 ± 2.62	5.68 ± 2.70		−2.562^**^
	T2	5.69 ± 2.65	5.86 ± 2.72	5.63 ± 2.62		1.854
	T3	5.70 ± 2.75	5.65 ± 2.82	5.72 ± 2.73		−0.545

Note*.* Values are presented as frequency (%) or mean ± standard deviation. ^*^p < *0.05,* ^**^p < *0.01*

† Statistically significant values based on adjusted standardized residuals are shown in bold italics.

Chi-squared tests revealed significant differences in sex (*p* = 0.031) and economic status (*p* < 0.001). Female participants reported significantly higher frequencies of COVID-19 diagnoses than did male participants (χ² = 4.658). Post-hoc analysis revealed significant results when adjusted residuals exceeded ±1.96. The non-diagnosed group had a significantly higher frequency of incomes below 1.5 million KRW, whereas the diagnosed group had significantly more individuals with incomes above 5 million KRW (adjusted residuals = 2.5, 3.8). However, the analysis revealed no significant differences in age or educational status.

The diagnosed group exhibited significantly higher severity of depression (*t* = 3.008, *p* = 0.003) and anxiety (*t* = 3.188, *p* = 0.001) than did the non-diagnosed group. Social isolation scores were analyzed longitudinally across three time points: T1 (2021), T2 (2022), and T3 (2023). At T1, participants diagnosed with COVID-19 (M = 5.34, SD = 2.62) reported significantly lower social isolation scores than did those who were not diagnosed with COVID-19 (M = 5.68, SD = 2.70) (*t* = −2.562, *p* = 0.008). No significant differences based on the diagnostic status were found at T2 and T3.

### Correlations between predictors

The present study sought to investigate the trajectory of social isolation levels from T1 to T3, focusing on variations among groups determined by the interaction of mental health variables and COVID-19 diagnosis. To explore the associations among mental health, COVID-19 diagnosis at T1, and the progression of social isolation from T1 to T3, a correlation analysis was performed (Table 2). Depression at T1 showed significant positive associations with anxiety at T1 (*r* = 0.735, *p* < 0.001) and social isolation at T1 (*r* = 0.402, *p* < 0.001), T2 (*r* = 0.304, *p* < 0.001), and T3 (*r* = 0.268, *p* < 0.001). Similarly, anxiety at T1 showed a significant positive association with social isolation at T1 (*r* = 0.311, *p* < 0.001), T2 (*r* = 0.226, *p* < 0.001), and T3 (*r* = 0.186, *p* < 0.001). In addition, social isolation showed strong correlations across time points, including T1 and T2 (*r* = 0.518, *p* < 0.001), T1 and T3 (*r* = 0.423, **p* *< 0.001), and T2 and T3 (**r* *= 0.425, *p* < 0.001).

**Table 2:** Bivariate correlations among depression and anxiety (2021) and social isolation (2021, 2022, 2023).

	Depression	Anxiety	Social isolation		
			Time 1	Time 2	Time 3
**Depression**	1				
**Anxiety**	0.735^***^	1			
**T1 Social isolation**	0.402^***^	0.311^***^	1		
**T2 Social isolation**	0.304^***^	0.226^***^	0.518^***^	1	
**T3 Social isolation**	0.268^***^	0.186^***^	0.423^***^	0.425^***^	1

* *p < 0.05,*

** *p < 0.01,*

*** *p < 0.001.*

### Model comparison

The analysis began by calculating the intraclass correlation coefficient (ICC) to assess the proportion of the total variance in social isolation scores attributable to individual differences. The ICC was 0.45, indicating that approximately 45% of the variance in the isolation levels was due to between-individual differences. This notable ICC value suggests that a considerable portion of the variation in social isolation can be explained by individual differences, justifying the use of a multilevel modeling approach.

Subsequently, a series of models were compared to determine the best-fitting model. The comparison began with the basic model (Model 1), which included only random intercepts without any predictors. This model serves as the baseline for evaluating the inclusion of additional terms. Next, a time-fixed effects model (Model 2) was introduced, assuming that the effect of time on social isolation was consistent across all individuals. To capture individual variability in the trajectory of social isolation over time, a random slope model (Model 3) was then tested, allowing the time slope to vary across individuals.

Regarding model selection criteria, the Random Slope Model had the lowest AIC. Although the BIC for the Random Slope Model was slightly higher than that for the Basic Model, the adjusted BIC (aBIC) suggested that the Random Slope Model provided the best fit, as it had the lowest aBIC value among the models tested. The adjusted BIC (aBIC) is particularly useful in model selection when dealing with mixed-effects models that involve hierarchical or multilevel data because it more accurately reflects the model’s complexity. In this study, the use of aBIC was crucial because it accounts for both the fixed and random effects for time, as well as the inherent variability between subjects [31]. The progressively lower AIC and aBIC values across the models suggested that the Random Slope Model with a three-way interaction effectively captured individual differences in isolation trajectories over time (Table 3).

**Table 3:** Model fit indices for social isolation.

	Basic Model (Model 1)	Time Fixed Effect Model (Model 2)	Random Slope Model (Model 3)
AIC	33294.04	33297.80	33287.42
BIC	33314.68	33325.31	33328.70
aBIC	33291.34	33294.19	33282.01

*Note.* AIC: Akaike Information Criterion; BIC: Bayesian Information Criterion; aBIC: adjusted BIC

### Multilevel analysis

A multilevel analysis was conducted to examine the effects of depression or anxiety severity and COVID-19 infection status on the trajectory of social isolation throughout the pandemic. To account for the strong correlation between depression and anxiety and to assess their unique contributions, the primary model simultaneously included two three-way interaction terms: depression × COVID-19 diagnosis × time and anxiety × COVID-19 diagnosis × time. To account for potential confounding and clarify the main effects, the model included the following two-way interaction terms as well: time × each mental health variable and COVID-19 diagnosis × each mental health variable. In addition, all main effects and key sociodemographic covariates, namely sex, age, education level, and household income, were included in the model.

The results indicated that individuals with no COVID-19 diagnosis (B = −0.382, *p* = 0.018), higher depression levels (B = 1.762, *p* < 0.001), being male (B = 0.210, *p* = 0.010), lower education (B = −0.137, *p* = 0.004), older age (B = 0.189, *p* < 0.001), and lower income (B = −0.288, *p* < 0.001) experienced greater social isolation at baseline (Table 4). Time and anxiety severity showed no significant main effects when imputing all predictors and interaction terms.

**Table 4:** Multilevel model analysis of social isolation.

Predictors	Estimate	*SE*	*t*	*p*	95% CI	
**Level 1**						
Time	0.011	0.034	0.347	0.729	−0.055	0.079
Anxiety at W1	0.322	0.190	1.699	0.089	−0.049	0.694
COVID-19 diagnosis at W1	−0.382	0.161	−2.373	0.018	−0.698	−0.067
Depression at W1	1.762	0.188	9.332	<0.001	1.392	2.132
Educational level	−0.137	0.048	−2.877	0.004	−0.230	−0.044
Gender	0.210	0.079	2.593	0.010	0.050	0.361
Age	0.189	0.027	7.030	<0.001	0.137	0.242
Income	−0.288	0.044	−6.523	<0.001	−0.374	−0.202
**Level 2**						
Anxiety at W1*Time	−0.095	0.081	−1.175	0.240	−0.252	0.063
Anxiety at W1* COVID19 diagnosis at W1	0.283	0.356	0.794	0.428	−0.415	0.981
Time* COVID19 diagnosis at W1	0.144	0.068	2.097	0.036	0.009	0.278
Time* Depression at W1	−0.267	0.080	−3.336	0.001	−0.424	−0.110
COVID19 diagnosis at W1* Depression at W1	−0.975	0.343	−2.839	0.005	−1.647	−0.303
**Level 3**						
Anxiety at W1*Time* COVID19 diagnosis at W1	−0.114	0.151	−0.750	0.454	−0.410	0.183
Depression at W1*Time* COVID19 diagnosis at W1	0.400	0.146	2.739	0.006	0.114	0.685

Note*.* Depression and anxiety severity scores were grand-mean-centered.

As shown in Table 4, significant two-way interactions were found between time and COVID-19 diagnosis at baseline, time and depression severity at baseline, and COVID-19 diagnosis and depression severity at baseline. In contrast, anxiety severity did not exhibit significant interaction effects with COVID-19 diagnosis or time.

To assess the robustness of these findings, we conducted sensitivity analyses by testing each three-way interaction term in separate models and found that the depression × COVID-19 diagnosis × time interaction remained statistically significant across all model specifications, whereas the anxiety × COVID-19 diagnosis × time interaction was not significant in any configuration. These findings suggested that the observed effect of depression was stable and not a result of model multicollinearity.

**Fig 1:**
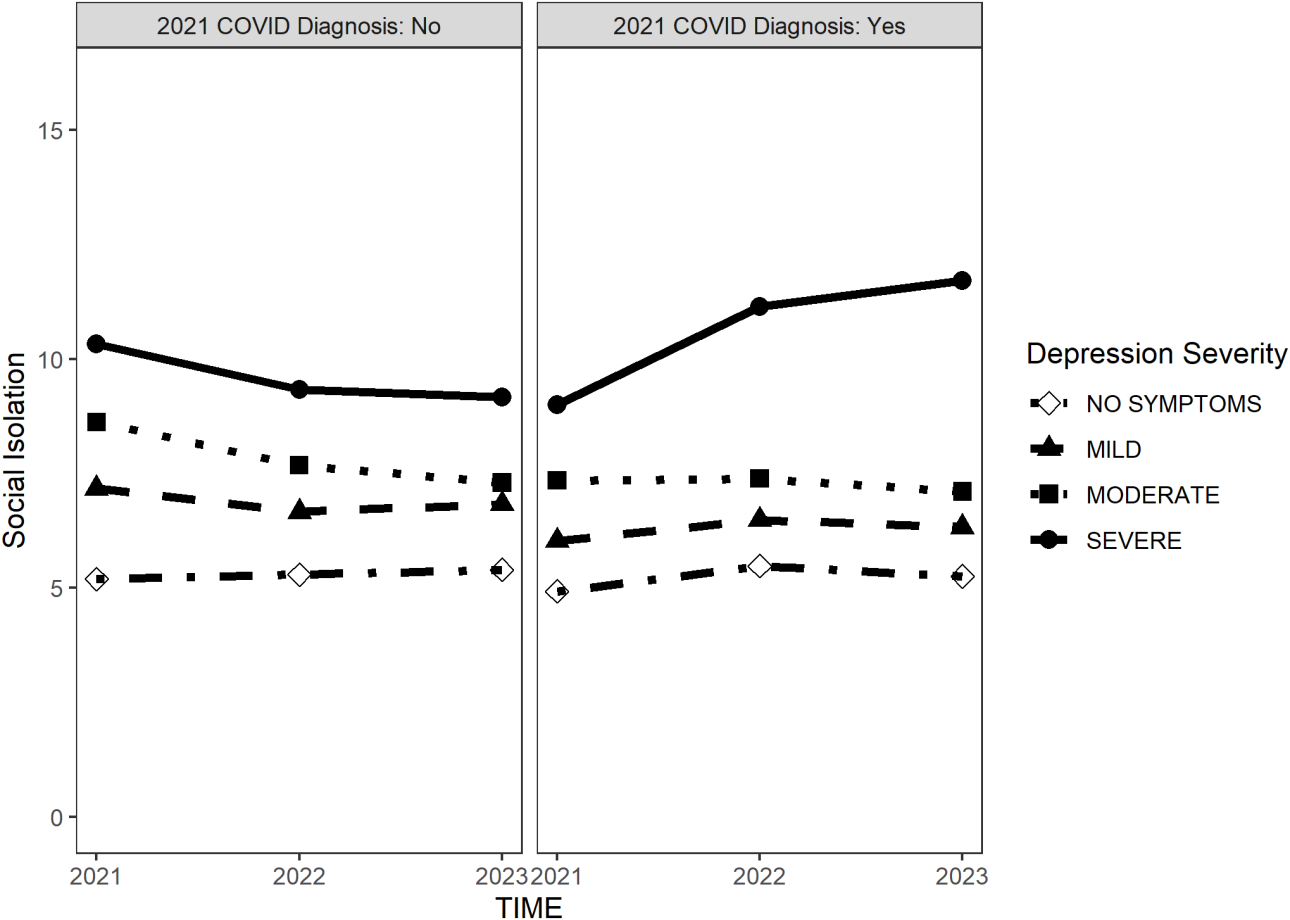
Three-way interaction between COVID-19 diagnosis, depressive severity, and time spent in social isolation.

Moreover, there was a significant three-way interaction between time, depression severity, and COVID-19 diagnosis (B = 0.400, *p* = 0.006), whereas the interaction among time, anxiety severity, and COVID-19 diagnosis was not significant. This suggests that the trajectory of social isolation was moderated by both depression severity and COVID-19 infection. Specifically, individuals with higher depression consistently experienced higher levels of social isolation, regardless of their COVID-19 diagnosis, over the 3 years. However, those with severe depression and a COVID-19 diagnosis experienced a significantly greater increase in social isolation over time than did those with severe depression but no COVID-19 diagnosis (Fig 1).

## Discussion

In this study, we examined longitudinal changes in social isolation over a 3-year period following the COVID-19 pandemic and investigated how initial psychological symptoms (depression and anxiety) and COVID-19 diagnosis predicted these changes. By focusing on baseline measures of mental health, we assessed how early psychological vulnerabilities can shape the long-term trajectories of social isolation. This approach allowed us to explore the enduring impact of initial mental health conditions on social outcomes in the context of a major public health crisis. In 2021, South Korea experienced fluctuating social distancing levels, ranging from Level 2 to the more stringent Level 4, which limited gatherings and marked the commencement of the vaccination campaign. Despite the continuation of vaccinations in 2022, confirmed cases exceeded 10 million, alongside the easing of outdoor mask mandates and adjustments to indoor requirements. By 2023, mask mandates were completely lifted, indicating a stabilization of infection rates.

The present study aimed to assess the initial impact of COVID-19 diagnosis during T1, amidst stringent social distancing measures. The selection of T1 as the primary independent variable was attributed to the high restriction levels and the initiation of national vaccination efforts during that time; this allowed for evaluation of the early effects of the pandemic amidst evolving public health measures. Multilevel analysis revealed a three-way interaction between time, COVID-19 diagnosis, and depression severity on social isolation change during the 3 years pandemic period, whereas there was no significant three-level interaction between anxiety, COVID-19, and time on social isolation.

The results of this study indicated that higher levels of depression and anxiety, lower educational attainment, being male, older age, and having no history of COVID-19 were associated with higher initial levels of social isolation. These findings are consistent with those of previous studies that identified depression as a key risk factor for social isolation. Specifically, higher levels of depression strongly predict increased social isolation, whereas greater anxiety correlates with higher isolation levels [17,22]. Additionally, demographic factors such as gender, age, education, and income play a significant role. For instance, men tend to experience more social isolation than do women across their lifespan, while lower educational attainment and older age are linked to increased isolation [32–34]. Personal finances and mental health were key predictors of loneliness and isolation before and during the pandemic [35]. However, in this study, anxiety severity and time were not significant predictors after controlling for the effects of other predictors and interaction terms. This highlights the need for a more comprehensive understanding of the interaction between these variables. In previous studies utilizing network analysis, depression was identified as a significant predictor of social isolation, and anxiety was found to indirectly influence social isolation through its impact on depression [36]. This demonstrates that depression has a stronger association with isolation than does anxiety, consistent with findings from previous research.

This study also identified a significant three-way interaction between depression severity, COVID-19 diagnosis in 2021, and time. Initially, individuals without a COVID-19 diagnosis experienced higher levels of social isolation; however, this trend shifted over time. Notably, individuals with severe depression who were diagnosed with COVID-19 in 2021 experienced a sustained increase in social isolation over time, whereas those with severe depression who were not infected did not. These findings suggest that COVID-19 is a significant risk factor for prolonged social isolation among people with severe depression.

Previous research supports these findings and demonstrates similar trends in social isolation and mental health during the pandemic. Similarly, a larger cross-sectional survey of 20,398 respondents across 101 countries revealed that 21% experienced severe loneliness during the pandemic, compared to 6% in the pre-pandemic period [35]. Social isolation has been associated with depression, anxiety, suicidal risk [37], and substance use disorders [38] during the pandemic. Collectively, these studies highlight the strong association between social isolation and mental health challenges during a pandemic.

Our findings also correspond with those of prior research on the mental health burden faced by COVID-19 survivors. Previous studies have shown that COVID-19 survivors showed substantially high rates of anxiety and depression. The prevalence of depressive disorder and anxiety disorder in a one-month follow-up after recovery in COVID-19 survivors was reported to be 31% and 42%, respectively [2], and 65.3% and 36%, respectively [10]. Wu et al. [39] reported that 20% and 14% of the participants showed moderate-to-severe depressive and anxiety symptoms at 4 weeks after discharge.

These findings suggest that the interplay between psychological factors (such as depression) and physical health (COVID-19) significantly influenced the trajectory of social isolation over time, even after controlling for three-way interaction terms between depression or anxiety, time, and COVID-19 diagnosis; two-way interaction terms between depression or anxiety and time and between depression or anxiety and COVID-19 diagnosis; baseline anxiety severity; depression severity; COVID-19 diagnosis; educational status; sex; age; and income.

### Strengths and limitations

The strength of this study is that it rigorously tested the variables by including both interaction terms and individual predictors in the same model to mitigate the effects of the strong correlation between anxiety and depression. The study also demonstrated that models with individual three-way interaction terms analyzed separately yielded the same results as models that included both interaction terms together. This confirms the robustness of the findings.

Another key strength of this study is its longitudinal design, which allows for a more nuanced understanding of how social isolation evolved during and after the pandemic and its relationship with depression, anxiety, COVID-19, and demographic characteristics. While extensive research has explored the relationships among social isolation, mental health, and their interactions with COVID-19, most studies utilized cross-sectional methodologies. The present study adopted a longitudinal approach, enabling a nuanced exploration of these dynamics over time. Our results not only corroborate previous findings but also deepen our comprehension by highlighting temporal changes and potential causal inferences within these relationships.

Furthermore, the current study controlled for various factors such as depression, anxiety, COVID-19 infection history, age, sex, educational status, and income. This multivariate approach facilitates a clearer identification of the independent effects of each variable on social isolation, as well as the complicated interactions among these factors.

Although most of the existing research on COVID-19 and social isolation has focused on older adults, this study included a broader age range, enabling the exploration of a more diverse population sample. This increases the generalizability of the findings to a wider demographic group.

However, this study had a few limitations. First, the non-significant interaction between anxiety severity, COVID-19 diagnosis in 2021, and time, after controlling for baseline anxiety and depressive severity, COVID-19 diagnosis, and other covariates, suggests that the relationship between anxiety and social isolation is less sensitive to the impact of COVID-19 over time than is the relationship between depression and social isolation. It is possible that while anxiety increased during the pandemic, as shown in previous studies [40], its effects on long-term social isolation may have been more variable or influenced by other factors such as demographic factors and depression. Therefore, the impact of anxiety may require further investigation in different contexts or over an extended period.

Second, the current study did not account for detailed information regarding the participants’ medical history, including any chronic conditions or the severity of their COVID-19 symptoms. Other variables beyond the predictors examined in this study could also contribute to social isolation. Future research should explore additional factors that may influence social isolation to provide a more comprehensive understanding.

Third, this study evaluated depression and anxiety only at baseline, limiting the ability to observe changes in psychological symptoms over time and their potential longitudinal bidirectional relationship with social isolation. Future studies should incorporate repeated mental health assessments for a more nuanced understanding of these dynamics.

Fourth, this study relied on self-reported measures, which may be subject to potential biases such as recall bias and social desirability bias. Although validated instruments and anonymous survey procedures were used to minimize these risks, the inherent limitations of self-reported data remain. Future research would benefit from incorporating objective behavioral or clinical indicators, as well as data from multiple sources, to improve the validity of the findings. In addition, to enhance generalizability and long-term relevance, future studies should include more diverse populations from different cultures and nationalities and utilize longitudinal designs with extended follow-up periods. Finally, as the data were collected within South Korea’s specific sociocultural and temporal boundaries during the COVID-19 pandemic, the generalizability of the findings to other countries with different cultures and public health responses may be limited.

## Conclusions

In conclusion, this study provides a detailed understanding of how mental health conditions influence social isolation throughout the pandemic by analyzing the interaction between depression, anxiety, and social isolation over time. This insight could inform the development of targeted interventions aimed at mitigating the long-term impact of social isolation. Individuals with severe depression who have been diagnosed with COVID-19 face an elevated risk of prolonged social isolation. This emphasizes the urgent need for early screening and intervention strategies to mitigate the long-term psychological and social effects of large-scale public health crises such as COVID-19.

## Supporting information

S1 File Three-way interaction between COVID-19 diagnosis, depressive severity, and time spent in social isolation. This represents the mean value shown in Figure 1.
(PDF)
