# Supplementary material for: Impact of depression, anxiety, and COVID-19 diagnosis on social isolation trajectories during the pandemic: A 3-year prospective cohort study
Source: PLoS One. 2025 Sep 10;20(9):e0330118. doi: 10.1371/journal.pone.0330118 (PMC12422492; doi:10.1371/journal.pone.0330118)
Supplement: S1 File — This represents the mean value shown in Figure 1. (PDF) [file pone.0330118.s001.pdf]

Figure 1. Three-way interaction between COVID-19 diagnosis, depressive severity, and time spent in social isolation.

COVID Diagnosis	Time	Depression Severity	Social Isolation Mean
No	2021	No Symptoms	5.19
	2022	No Symptoms	5.29
	2023	No Symptoms	5.40
	2021	Mild	7.17
	2022	Mild	6.66
	2023	Mild	6.83
	2021	Moderate	8.62
	2022	Moderate	7.68
	2023	Moderate	7.30
	2021	Severe	10.33
	2022	Severe	9.33
	2023	Severe	9.17
Yes	2021	No Symptoms	4.93
	2022	No Symptoms	5.48
	2023	No Symptoms	5.25
	2021	Mild	6.03
	2022	Mild	6.48
	2023	Mild	6.33
	2021	Moderate	7.35
	2022	Moderate	7.39
	2023	Moderate	7.10
	2021	Severe	9.00
	2022	Severe	11.14
	2023	Severe	11.70
